# Supplementary material for: Use of a continuous single lead electrocardiogram analytic to predict patient deterioration requiring rapid response team activation
Source: PLOS Digit Health. 2024 Oct 24;3(10):e0000465. doi: 10.1371/journal.pdig.0000465 (PMC11500862; doi:10.1371/journal.pdig.0000465)
Supplement: S1 Appendix — (DOCX) [file pdig.0000465.s001.docx]

## S1 Appendix: Steps taken for conducting the case-control study.

1. Start from all patient-encounters with ECG monitoring data.
2. Remove patients already included the present study.
3. Keep patients who are 18 years old or older at the start time of ECG monitoring.
4. Keep if no contraindications.
5. Remove patients that have none of the principal diagnoses of the patients in the present study.
6. Remove patients with RRT or code events regardless of whether they were during ECG monitoring or not
7. Randomly shuffle the RRT cases to go through and find a match for
8. For each of the RRT cases
   1. Find those with same sex, age (+/- <6 years), and unit type as the given case
   2. Keep patients if they have at least one principal diagnosis that the given case’s patient has.
   3. Choose the control case whose ECG monitoring length is the closest to the given case’s.
      1. If there are 2 or more control cases according to meeting this criterion, randomly choose one.
      2. If the control case was already matched to another RRT case, then choose the next best match.
9. Assign an “event time” to each of the chosen control cases according to its matched RRT cases such that the events happen proportionately at the same time during ECG monitoring.
